# Apelin-13 in septic shock: effective in supporting hemodynamics in sheep but compromised by enzymatic breakdown in patients

**DOI:** 10.1038/s41598-021-02087-4

**Published:** 2021-11-23

**Authors:** David Coquerel, Julie Lamoureux, Frédéric Chagnon, Kien Trân, Michael Sage, Etienne Fortin-Pellerin, Eugénie Delile, Xavier Sainsily, Justin Fournier, Audrey-Ann Dumont, Mannix Auger-Messier, Philippe Sarret, Eric Marsault, Jean-Paul Praud, Tamàs Fülöp, Olivier Lesur

**Affiliations:** 1 Centre de Recherche Clinique du CHUS, Faculté de Médecine Et Des Sciences de La Santé, Université de Sherbrooke, Sherbrooke, QC Canada; 2 Département de Médecine, Service de Cardiologie, Faculté de Médecine Et Des Sciences de La Santé, Université de Sherbrooke, Sherbrooke, QC Canada; 3 Centre de Recherche Sur Le Vieillissement, Faculté de Médecine Et Des Sciences de La Santé, Université de Sherbrooke, Sherbrooke, QC Canada; 4 Départements de Pédiatrie Et de Pharmacologie/Physiologie, Faculté de Médecine Et Des Sciences de La Santé, Université de Sherbrooke, Sherbrooke, QC Canada; 5 Département de Pharmacologie-Physiologie, Faculté de Médecine Et Des Sciences de La Santé, Université de Sherbrooke, Sherbrooke, QC Canada; 6 Institut de Pharmacologie de Sherbrooke (IPS), Faculté de Médecine Et Des Sciences de La Santé, Université de Sherbrooke, Sherbrooke, QC Canada; 7 Unité Des Soins Intensifs Médicaux Et Service de Pneumologie, Faculté de Médecine Et Des Sciences de La Santé, Université de Sherbrooke, 3001 12th Avenue Nord, Sherbrooke, QC J1H 5N4 Canada

**Keywords:** Biochemistry, Physiology, Cardiology, Diseases, Medical research

## Abstract

Sepsis is a prevalent life-threatening condition related to a systemic infection, and with unresolved issues including refractory septic shock and organ failures. Endogenously released catecholamines are often inefficient to maintain blood pressure, and low reactivity to exogenous catecholamines with risk of sympathetic overstimulation is well documented in septic shock. In this context, apelinergics are efficient and safe inotrope and vasoregulator in rodents. However, their utility in a larger animal model as well as the limitations with regards to the enzymatic breakdown during sepsis, need to be investigated. The therapeutic potential and degradation of apelinergics in sepsis were tested experimentally and in a cohort of patients. (1) 36 sheep with or without fecal peritonitis-induced septic shock (a large animal experimental design aimed to mimic the human septic shock paradigm) were evaluated for hemodynamic and renal responsiveness to incremental doses of two dominant apelinergics: apelin-13 (APLN-13) or Elabela (ELA), and (2) 52 subjects (33 patients with sepsis/septic shock and 19 healthy volunteers) were investigated for early levels of endogenous apelinergics in the blood, the related enzymatic degradation profile, and data regarding sepsis outcome. APLN-13 was the only one apelinergic which efficiently improved hemodynamics in both healthy and septic sheep. Endogenous apelinergic levels early rose, and specific enzymatic breakdown activities potentially threatened endogenous apelin system reactivity and negatively impacted the outcome in human sepsis. Short-term exogenous APLN-13 infusion is helpful in stabilizing cardiorenal functions in ovine septic shock; however, this ability might be impaired by specific enzymatic systems triggered during the early time course of human sepsis. Strategies to improve resistance of APLN-13 to degradation and/or to overcome sepsis-induced enzymatic breakdown environment should guide future works.

## Introduction

Sepsis is a dysregulated host response to infection that includes a combination of heterogeneous and inflammatory events^1^. Septic shock with multiple organ failure is a major issue of sepsis resulting in elevated death rates. Associated cardiovascular dysfunction is one of the three main acute organ dysfunctions related with short-term mortality^2^. Endogenous stress-released vasopressor catecholamines spilled into the bloodstream during the initial inflammatory storm in septic shock are ineffective. Those have reduced signaling capacity because they are oxidized by the reactive oxygen species-rich environment and associated with diminished downstream adrenergic receptor sensitivity^3,4^. Strategies aimed at improving the catecholamine-to-adrenergic receptor ratio and potentially countering this ineffectiveness with exogenous unoxidized catecholamines are often similarly inefficient, leading to sympathetic overstimulation and ultimately harmful effects^5–7^.

Alternative noncatecholaminergic therapies therefore represent new avenues for medical interventions in sepsis. The apelin system, mediator of cardiovascular control, is one such option, and it has been reported to have valuable cardiovascular support properties (i.e., cardiac inotropy, vasoregulation, fluid homeostasis, and renal protection) in experimental and clinical research^8^. The recognized endogenous agonists (hereinafter, “apelinergics”) of the only known specific G protein-coupled receptor (GPCR) in the apelin system (named APJ; gene symbol, *APLNR*) are (1) apelins (APLNs), namely, the APLN-36, APLN-17, APLN-13, and APLN-12 amino acid isoforms, of which the pyroglutamate-modified form of APLN-13 (pyr-APLN-13) is the dominant form in the cardiovascular system^9^; and (2) Elabela (ELA) (32 amino acids), which has also been described as an important regulator of cardiovascular function^10^. Infusion of these molecules has been shown to produce cardioprotective effects and to stabilize hemodynamics in rodent models of heart failure^11,12^ and sepsis-induced myocardial dysfunction^13^, promoting improved survival and exhibiting a good safety profile^14^. However, reported spontaneous increases of APLN-13 levels in the blood have been modest in the acute phase of heart failure^15^ as well as in cardiovascular dysfunction induced by sepsis^16^, suggesting inadequate reactivity of the apelin system or accelerated degradation/clearance of effective peptides. In sepsis, knowledge is lacking on how this apelin system is reactive; if it has therapeutic potential in larger animal models, and what is potentially affecting the homeostasis of the apelinergics in this context.

Indeed, although nothing is known about the enzymatic breakdown pathways specific to ELA, apelins are recognized to be small peptides that are very susceptible to degradation and corresponding loss of function^17^. More specifically, members of the renin-angiotensin (RAS) and kallikrein–kinin (KKS) systems, i.e., angiotensin-converting enzyme type II (ACE2)^18,19^, kallikrein (KLK1)^20^, and neprilysin (NEP)^21,22^, can degrade native apelin isoforms and affect bioavailability, with varying impacts on activity. Consequently, e.g., through increased apelin levels with consecutive enhanced endogenous homeostasis and hemodynamic responsiveness, inhibition of the RAS system could lead to better shock outcomes. De facto, chronic use of a RAS inhibitor for cardiovascular protection- by patients has been reported to improve septic shock outcomes^23,24^.

The main hypotheses of this work were that (i) exogenously infused apelinergics would ameliorate the initial hemodynamic instability in a large animal model of acute experimental septic shock and that (ii) in humans, the endogenous apelin system is reactive in early septic shock but potentially inactivated by enzymatic degradation, which can compromise its hemodynamic impact. To test these hypotheses, (1) we evaluated the hemodynamic dose–response to apelinergic infusion in a sheep model of septic shock induced by fecal peritonitis (FP), and (2) we assessed the reactivity of the endogenous apelin system and its related enzymatic degradation pathway in patients with acute septic shock. A main finding was that the hemodynamic supporting activity of pyr-APLN-13 further confirmed during sepsis could be environmentally compromised by specific activated breakdown systems. This will pave the way to implement optimization strategies for advancing the apelin system as a target of therapeutic intervention in sepsis.

## Results

### Human elabela (ELA) exhibits distinctive signaling from apelin-13 (APLN-13)

Assessed by competitive radioligand binding experiments, the affinities of human APLN-13 and human ELA for sheep APJ were similar (Additional file: Fig. S1A). However, BRET assays revealed that ELA binding to sheep APJ exhibited lower potency in G_αi1_ activation and lower potency and efficacy in β-arrestin-2 recruitment than APLN-13 binding (Additional file: Fig. S1B and C).

### Apelin-13 (APLN-13) and Elabela (ELA) display distinctive and differential hemodynamic signatures in healthy sheep

**Figure 1:**
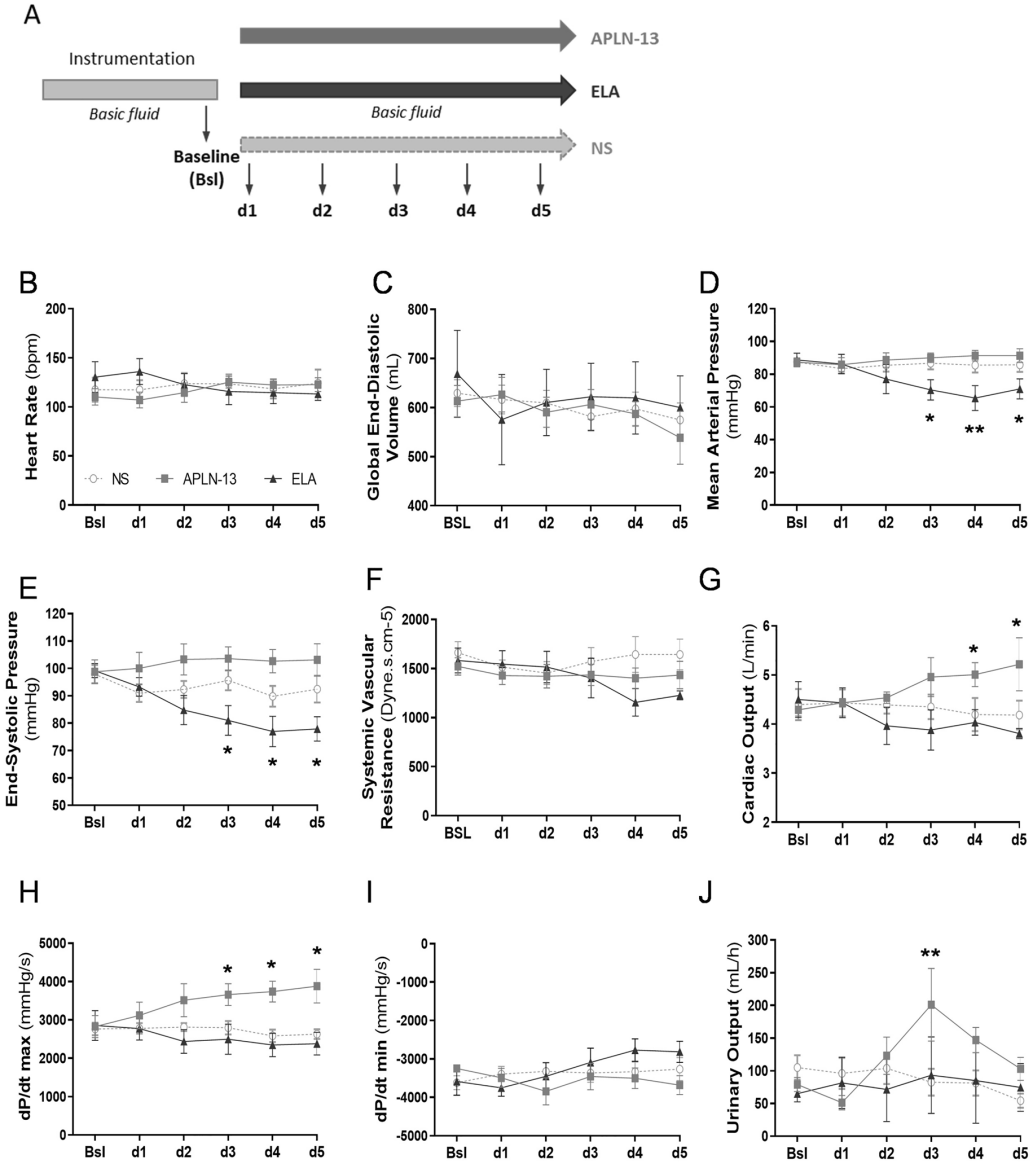
Hemodynamic signatures and diuretic impacts of apelin-13 (APLN-13) and Elabela (ELA) are distinctive and differential in healthy sheep. (**A**) Study Design: Sheep were maintained under general anesthesia and mechanical ventilation. Following instrumentation and clinical stabilization, displayed data were recorded at baseline (bsl) and following infusion of 20 min-incremental doses of APLN-13 or ELA (d1: 0.025; d2: 0.25; d3: 2.5; d4: 6.25; and d5: 12.5 nmol/kg/h) vs. normal saline (NS). Basal fluid delivery: Ringer Lactate (RL, 3 mL/kg/h) and 5% D-glucose (2 mL/kg/h). See “Methods” section for details. (**B–I**) Heart rate, global end-diastolic volume, mean arterial pressure; end-systolic pressure; systemic vascular resistance; cardiac output, dP/dt max and dP/dt min were assessed by PiCCO-Volef thermodilution or left ventricular catheterization. Above or from 2.5 nmol/kg/h, APLN-13 enhanced cardiac output and dP/dt max whereas ELA decreased mean arterial pressure and end-systolic pressure. (**J**) Urinary output was measured by percutaneous bladder catheterization. APLN-13 increased urine output at 2.5 nmol/kg/h. All results are expressed as the mean ± SEM (n = 6/group). Statistical analyses for quantitative variables were performed with a paired Student’s *t*-test (normally distributed variables) or a Mann Whitney U test (nonnormally distributed variables). Dose–response and time-course analyses were performed with repeated measures two-way ANOVA followed by Tukey’s multiple comparison test. *p < 0.05, **p < 0.01 vs. normal saline (NS)-infused sheep.

Experimental design for hemodynamic monitoring in healthy sheep is presented in Fig. 1A. Monitored with PiCCO-technology, heart rate (HR) and global end-diastolic volume (GEDV) (Fig. 1B,C) as well as the mean pulmonary arterial pressure (MAP) (data not shown) were unchanged in response to APLN-13 or ELA infusions. At 2.5 nMol/kg/h and greater concentrations, human ELA reduced significantly MAP (d3: − 20.9 ± 5.1%, p = 0.044; d4: − 26.2 ± 8.5%, p = 0.026; d5: − 20.3 ± 5.2%, p = 0.045; all vs. baseline), with a maximum decrease at 6.25 nMol/kg/h compared to that with normal saline (NS) and APLN-13 (ELA: 65.4 ± 8.1 mmHg; NS: 85.6 ± 4.2, p = 0.0006; APLN-13: 91.3 ± 2.9, p = 0.013) (Fig. 1D); end-systolic pressure was similarly affected (Fig. 1E) but systemic vascular resistance was not significantly impacted (Fig. 1F). Conversely, APLN-13 significantly improved LV systolic function as shown by enhanced cardiac output (CO) at 6.25 and 12.5 nMol/kg/h and dP/dt max from 2.5 to 12.5 nMol/kg/h (d3: + 44 ± 12%, p = 0.044; d4: + 47 ± 13%, p = 0.006; d5: + 39 ± 13%, p = 0.011; all vs. baseline) (Fig. 1G,H) but did not impact LV relaxation as shown by unchanged dP/dt min (Fig. 1I). Finally, an increase in urinary output was noted with APLN-13 at 2.5 nMol/kg/h (p = 0.045 and p = 0.009 vs. ELA and NS, respectively) (Fig. 1I).

### Apelin-13 (APLN-13) improves cardiorenal axis function in an ovine model of polymicrobial peritonitis mimicking human septic shock

**Figure 2:**
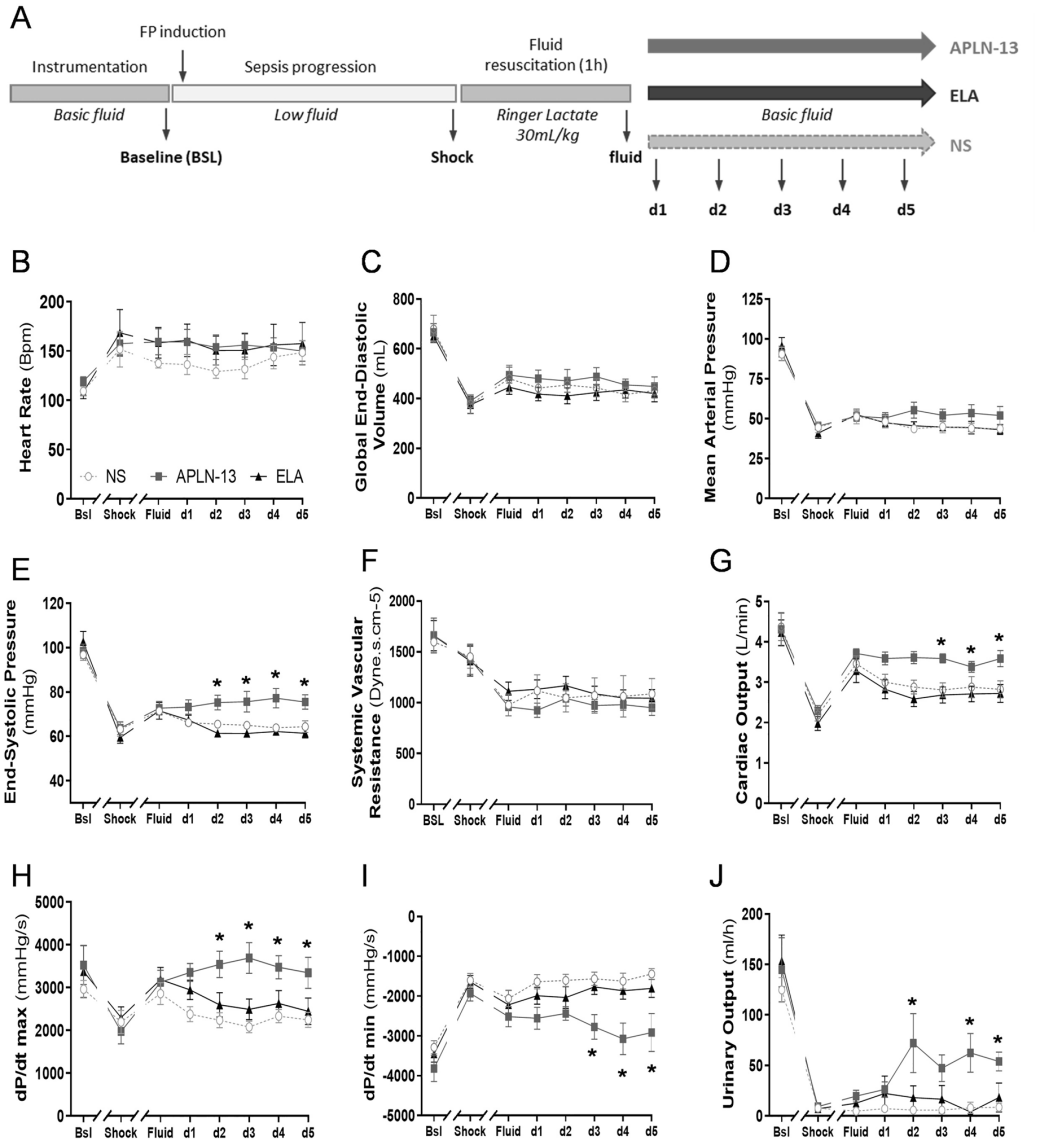
Apelin-13 (APLN-13) improves cardiorenal axis function in an ovine model of fecal peritonitis (FP). (**A**) Study Design: Sheep were first prepared as described in Fig. 1. After baseline assessment, fecal peritonitis was induced by intraperitoneal injection of a stool slurry (2 g/kg) and five criteria of shock must be met before fluid resuscitation challenge (RL 30 mL/kg for 1 h) and start of infusions of 20 min-incremental doses of APLN-13 or Elabela (ELA) vs. normal saline (NS), as described in Fig. 1. See “Methods” section for details. Septic shock was achieved 251 ± 15 min after FP induction. (**B–I**) Heart rate, global end-diastolic volume, mean arterial pressure; end-systolic pressure; systemic vascular resistance; cardiac output, dP/dt max and dP/dt min were assessed by PiCCO-Volef thermodilution or left ventricular catheterization at the baseline (Bsl), shock (Shock), and fluid resuscitation challenge (Fluid) time points and for d1 to d5 corresponding to increasing apelinergic doses. Following fluid resuscitation, APLN-13 maintained cardiac output and increased end-systolic pressure from 0.25 nmol/kg/h. APLN-13 also enhanced dP/dt max along with decreased dP/dt min from 0.25 nmol/kg/h and 2.5 nmol/kg/h, respectively. (**J**) Urinary output was measured by percutaneous bladder catheterization. APLN-13 increased urinary output from 0.25 nmol/kg/h. All results are expressed as the mean ± SEM (n = 6/group). Statistical analyses for quantitative variables were performed with a paired Student’s *t*-test (normally distributed variables). Dose–response and time-course analyses were performed with repeated measures two-way ANOVA followed by Tukey’s multiple comparison test. *p < 0.05 vs. normal saline (NS)-infused sheep.

Experimental design for hemodynamic monitoring in sheep with FP-induced acute septic shock is presented in Fig. 2A. Shock criteria were met 251 ± 15 min after FP induction and accompanied by several ensuing disorders (i.e., increased, lactate, troponin T (Tn T), cortisol and arginine-vasopressin (AVP) circulating levels as well as KIM-1 urinary levels or reduced ScvO2, base excess, creatinine clearance or oxygen consumption (VO_2_)) (Table 1). The average sheep Sequential Organ Failure Assessment (SOFA) score was 10.1 ± 1.5.

**Table 1:** Additional main biological parameters before and after FP-induced shock in sheep.

	Baseline	Shock		p-values
Lactate (mmol/L) *	0.8 (0.5–0.9)	2.2 (2–2.4)		< 0.0001
ScvO_2_ (%) ^†^	81.9 ± 4.7	64.1 ± 5.9		< 0.0001
Base excess (mmol/L) ^†^	8.1 ± 3.3	2.5 ± 4.1		< 0.0001
Tn T (pg/mL) *	154.5 (137.5–191.1)	168.5 (151.0–194.8)		0.002
Cortisol (ng/mL) ^†^	292.8 ± 198.4	3479 ± 2175		< 0.0001
AVP (pg/mL) ^†^	16.6 ± 5.3	18.8 ± 7.7		0.015
Urinary KIM-1 (pg/mL) ^†^	75.9 ± 25.1	102.7 ± 31.7		0.022
Creatinine clearance (mL/min) ^†^	147.2 ± 70.7	1.8 ± 1.7		< 0.0001
EVLW (mL) ^†^	413.1 ± 93.5	502.9 ± 104.6		0.011
VO_2_ (mL/min) ^†^	13.2 ± 3.6	11.1 ± 2.3		0.01

All data are expressed as the median [25–75 interquartile range] ^⁎^ or mean ± SD ^†^, as appropriate, for n = 18 sheep. Variation was compared with a paired *t*-test or the Mann Whitney U test.

*ScvO*_*2*_ central venous oxygen saturation, *Tn T* troponin T, *AVP* arginine vasopressin, *KIM-1* kidney injury molecule-1, *EVLW* extravascular lung water, *VO*_*2*_ oxygen consumption.

Sustained translocation of gram-negative bacilli (*Serratia marcescens, Klebsiella oxytoca, Citrobacter freundii,* and *Pseudomonas aeruginosa*) was revealed by blood culture performed one and two hours after FP induction (data not shown).

The values of almost all monitored hemodynamic parameters, except HR, decreased with FP, but incremental doses of ELA failed to alleviate these decreases in septic sheep (Fig. 2B–J). In contrast, APLN-13 (2.5 to 12.5 nMol/kg/h) stabilized fluid-induced CO restoration (CO: 2.5 nMol/kg/h; APLN-13: 3.61 ± 0.37 L/min; NS: 2.88 ± 0.42, p = 0.045; ELA: 2.71 ± 0.41, p = 0.012) and significantly enhance LV end-systolic pressure without effects on HR, GEDV, MAP or systemic vascular resistance (SVR) (Fig. 2B–G). APLN-13 also improved left ventricle (LV) contractility/relaxation (*i.e.*, dP/dt max: maximum response observed at 2.5 nMol/kg/h; + 19.2 ± 7.6%; dP/dt min maximum response observed at 6.25 nMol/kg/h, + 22.2 ± 6.4% (Fig. 2H,I). However, neither APLN-13 nor ELA infusion impacted the endpoint circulating levels of lactate, cortisol, and Tn T (Additional file: Fig. S2A to C). APLN-13 increased urinary output at a dosage of 0.25 nMol/kg/h and optimized creatinine clearance without impacting blood AVP levels (Fig. 2J & Additional file: Fig. S3D to F).

**Figure 3:**
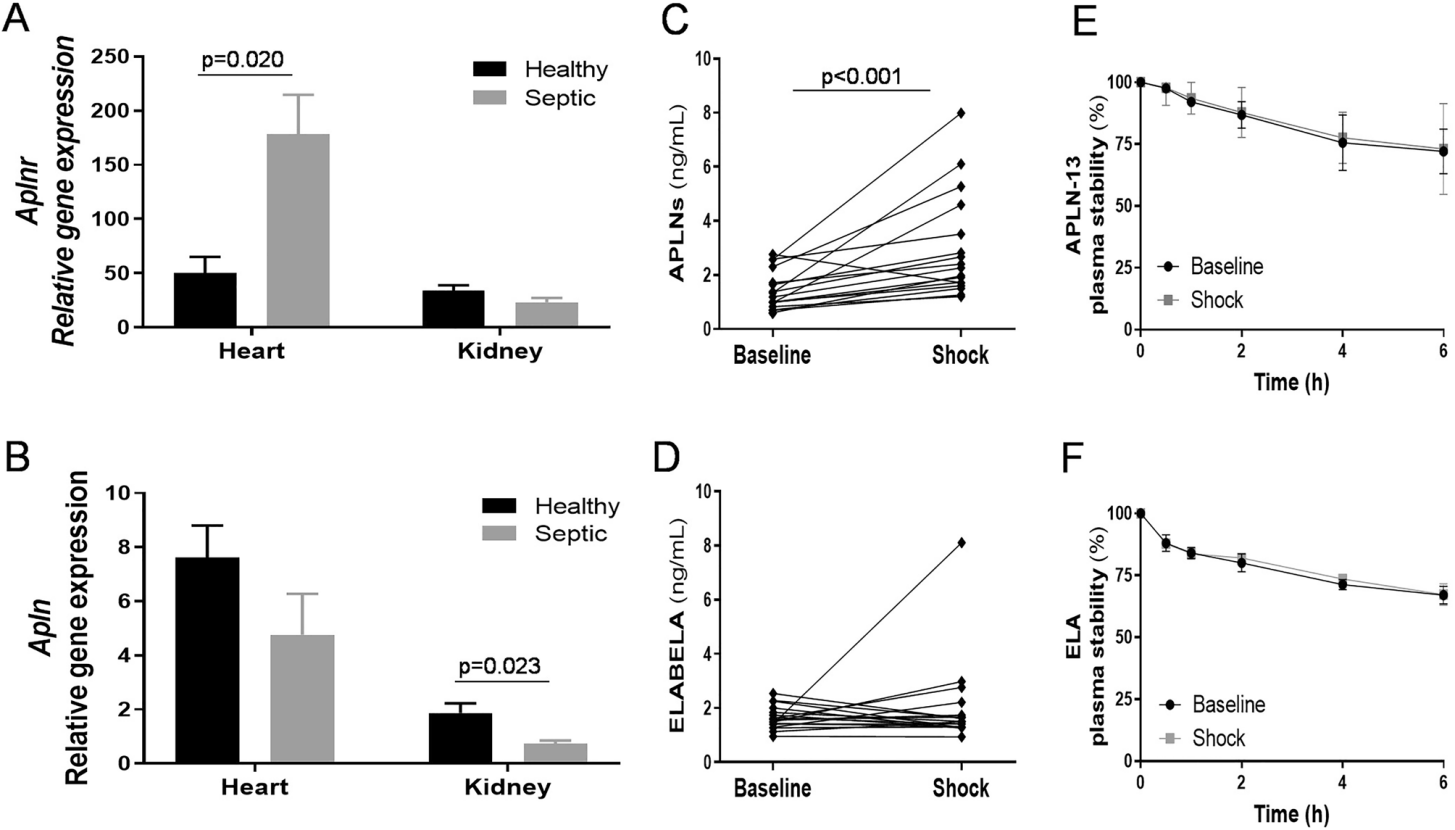
Ovine fecal peritonitis (FP) with septic shock activates the endogenous apelin system without inducing plasma peptide instability. (**A,B**) Ribonucleic acids were extracted from tissues and real-time PCR was performed. Heart and kidney apelin receptor -*Aplnr*- and apelin -*Apln*- mRNA expressions in healthy (black bar, n = 6) and septic sheep (gray bar, n = 6). The results are expressed as the mean ± SEM. FP induced increased expression of APJ in the heart but decreased expression of APLN in kidneys. (**C,D**) EDTA-preserved blood samples from the ovine FP model were used to perform specific enzymatic immunoassays (after extraction for ELA determination). APLN (all isoforms and moieties) and Elabela (ELA) plasma levels at the baseline and shock time points (individual data plots display, n = 18). (**E,F**) Stability of APLN-13 and ELA exogenously added to sheep plasma samples collected at the baseline and shock time points (n = 18). Stability was expressed as the residual percentage of non-degraded peptides. The results are expressed as the mean ± SEM. There was no obvious accelerated degradation of the two peptides. Statistical analyses were performed with a paired Student’s *t*-test (normally distributed variables) with exact p values for (**A,B**) and p < 0.001 for (**C**).

FP induction triggered activation of the endogenous ovine apelin system, with (i) increased APJ mRNA expression in the heart but decreased APLN-13 mRNA expression in the kidneys (Fig. 3A,B) and (ii) enhanced circulating levels of APLNs but not ELA (Fig. 3C,D). Notably, these apelinergics exhibited biochemical stability in sheep plasma (Fig. 3E,F).

### The apelinergic release-degradation network is dysregulated in early sepsis/septic shock

Thirty-three out of 45 eligible patients with sepsis agreed to participate in this study (9 were excluded because informed consent was refused or not obtained, and 3 were excluded because of unavailability of the recruitment staff). Calculated and effective outcomes, types of ICU admission, sepsis categories, sources of infections, and cyto-biochemical characteristics are shown in Table 2.

**Table 2:** General characteristics of the subjects enrolled in the study cohort.

	Patients diagnosed with sepsis n = 33	Healthy volunteers n = 19	*p*-values
Age (years) ^⁎^	68 (57.5–73)	71.5 (64–76.5)	*0.086*
Sex, male/female, n	20/13	7/12	*0.150*
APACHE II score^⁎^	26 (20–35)	N/A	*ND*
SOFA score ^⁎^	8 (5–11)	N/A	*ND*
Rate of in-hospital death, n (%)	9 (27)	N/A	*ND*
**Types of admission, n (%)**			*ND*
Medical	27 (82)	N/A	*ND*
Emergency surgery	6 (18)	N/A	*ND*
**Sepsis category, n (%)**			*ND*
Sepsis/septic shock	21 (64)	N/A	*ND*
Refractory septic shock	12 (36)	N/A	*ND*
**Types of infection, n**			
Pneumonia	13	N/A	*ND*
Urosepsis	8	N/A	*ND*
Fasciitis	5	N/A	*ND*
Cholangitis	2	N/A	*ND*
Mediastinitis	2	N/A	*ND*
Peritonitis	2	N/A	*ND*
Colitis	1	N/A	*ND*
Esophageal-pleural perforation	1	N/A	
Blood PMN counts (10^9^/L)	20.6 ± 3.3	10.1 ± 1.5	** < *0.0001‡***
Blood band cells (%) ^⁎^	1.2 (0.6–3.6)	0.05 (0–0.125)	** < *0.0001‡***
PMN CD64 expression (MFI) ^⁎^	407 (271–560)	179 (159–246)	** < *0.0001‡***
Pentraxin 3 (ng/mL) ^⁎^	8 (6–20)	2 (1–3)	***0.032‡***
Lactate (mmol/L) ^†^	4.55 ± 3.7	N/A	*ND*
Plasma cortisol level (nmol/L) ^†^	1176 (774–1750)	N/A	*ND*

Data are expressed as the median [25–75 interquartile range] or mean ± SD ^†^, as appropriate. The Mann–Whitney U test was used to compare age, and Fisher’s exact test was used for contingency in sex-based comparisons. Biological data were analyzed by Student’s *t*-test or the Mann–Whitney U test. There were four, five and two missing data points for CD64, PTX3, and cortisol measurements, respectively.

*N/A* not applicable, *ND* not determined, *APACHE II* Admission Acute Physiology and Chronic Health Evaluation II, *SOFA* Sequential Organ Failure Assessment, *PMN* polymorphonuclear neutrophil, *MFI* mean fluorescence intensity.

**Figure 4:**
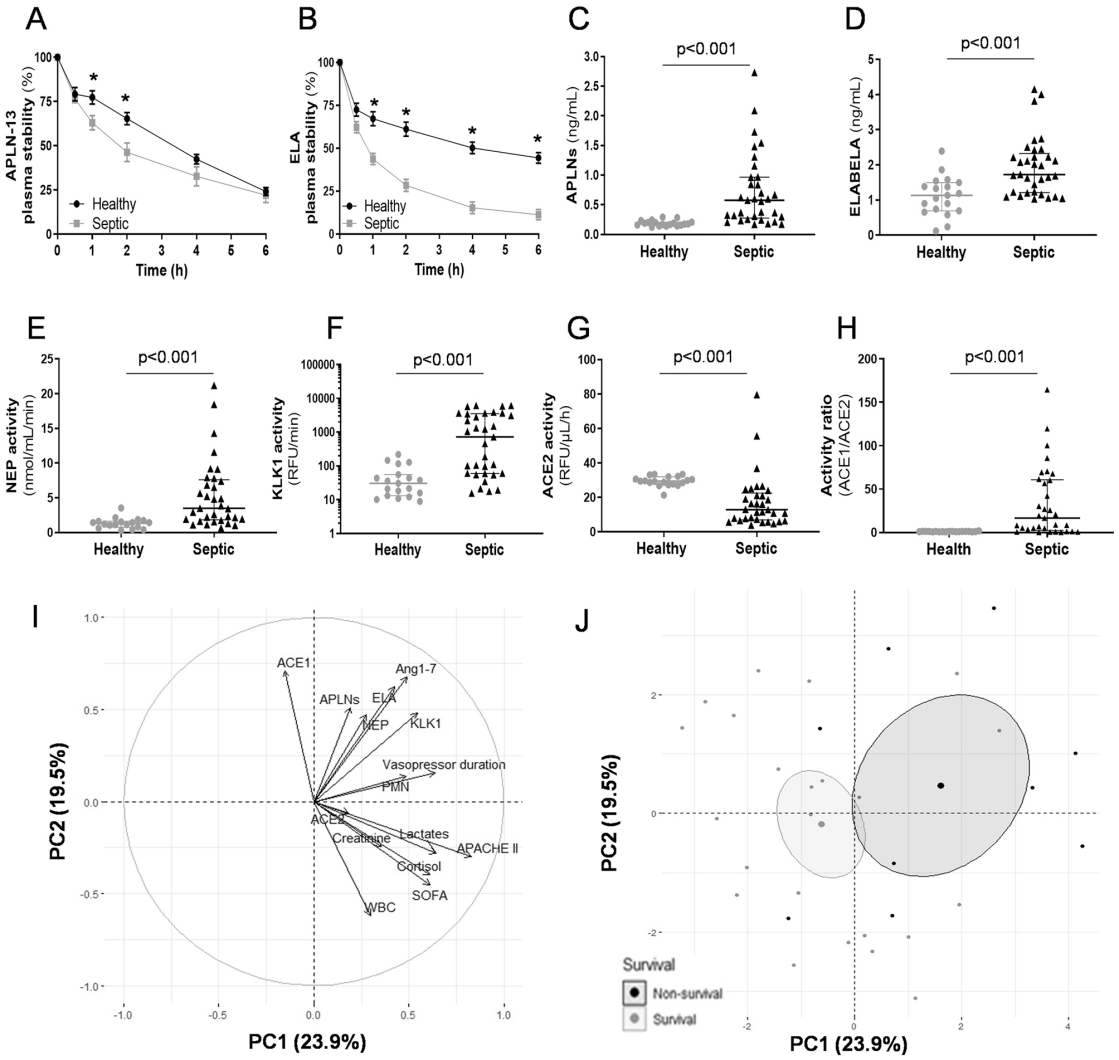
Acute septic shock dysregulated the apelinergic release-degradation rollover and was associated to the activation of specific enzymatic breakdown activities and worsened outcome impacts. A cohort of 33 patients with acute sepsis/septic shock (Sepsis) was included in the study and compared to 19 age-matched healthy volunteers (Healthy) for: (i) assessment of plasma peptide stability; (ii) apelin (APLN) and Elabela (ELA) plasma level determination; (iii) measurement of specific enzymatic breakdown activities; and iv) analyses of the statistical associations between biological; clinical and outcome data. (**A,B**) A randomly selected subset of Sepsis (n = 22) and Healthy (n = 10) plasma were assayed for APLN-13 and ELA stability. The results are expressed as the mean ± SEM. After 1 h incubation, APLN-13 -at two time-points- but also ELA – at four time-points- were more degraded by plasma from septic patients (Sepsis)., *p < 0.05 compared with time-matched values. (**C,D**) Plasma levels of APLNs (all isoforms and moieties) and ELA. Data are displayed as the median [25–75 interquartile range]. Sepsis vs. Healthy induced a higher magnitude of APLN vs. ELA increased levels in bloodstream. (**E–G**) Plasma activities of neprilysin (NEP), kallikrein (KLK1), and angiotensin-converting enzyme 2 (ACE2). (**H**) ACE1/ACE2 enzymatic activity ratio (individual data plots display). Sepsis vs. Healthy enhanced NEP; KLK1; and ACE1/ACE2 enzymatic activity ratio (with decreased ACE2 activity). (**I**) Correlation circle graph of 15 variables related to sepsis outcome from 33 patients and to the apelinergic release-degradation system. Two PCs (PC1 and PC2) were identified in a principal component analysis (PCA), explaining 43% of the variation in the dataset. (**J**) Individual dispersion graph of PCA split by survival status. The midpoint of the concentration ellipses is representative of the gravity center for each modality of survival. Statistical analyses for quantitative variables were performed with a Mann–Whitney U test (nonnormally distributed variables) for panels (**C–H**).

The plasma stability of exogenously added APLN-13 and ELA was lower in septic plasma than in healthy plasma in vitro, suggesting enhanced degradation in the sepsis environment. The ELA half-life was shortened to a greater extent and reached more rapidly than that of APLN-13 (APLN-13, sepsis: 143 ± 20 min, healthy volunteers: 194 ± 15 min, p = 0.045; ELA, sepsis: 69 ± 12 min, healthy volunteers: 258 ± 45 min, p = 0.003) (Fig. 4A,B). Nevertheless, septic plasma exhibited higher levels of APLNs and ELA (Fig. 4C,D) and lower ACE2 activity but higher ACE1 (data not shown), NEP, and KLK1 enzymatic activities than plasma from healthy volunteers (Fig. 4E–G). The ACE1/2 activity ratio was thus further enhanced in septic plasma (Fig. 4H) and associated with patient negative outcome (nonsurvivors, n = 10: 62.1 [6.3–77.9] vs. survivors, n = 23: 8.8 [0.7–26.9], p = 0.035). In addition, the decreased APLN-13 half-life was associated with increased NEP and KLK1 enzymatic activities (Additional file: Fig. S3A and B). Moreover, higher NEP but not ACE2 activity was associated with higher circulating Angiotensin 1–7 (Ang1-7) levels, suggesting this could be an alternative pathway during sepsis. (Additional file: Fig. S3C).

Principal component analysis (PCA) was performed on 15 intercorrelated continuous variables related to sepsis outcomes and apelin system metabolism, revealing two principal subspaces (PC1 and PC2) accounting for 19.46 and 23.94%, respectively (Fig. 4I). PCA showed a strong association among the following variables: arterial lactate levels, circulating creatinine and cortisol levels, and Acute Physiology and Chronic Health Evaluation (APACHE) II and SOFA scores, which altogether defined the PC1 axis. A second relationship among circulating apelin, ELA, and Ang1-7 levels and ACE1, KLK1, and NEP activities defined the PC2 axis. In an individual dispersion graph, sepsis nonsurvivors were widely distributed in the positive area of the PC1 and PC2 axes, suggesting that worse sepsis outcomes could have been influenced by the profile of the apelinergic release-degradation network in critically ill patients (Fig. 4J).

## Discussion

The specific aim of this work was to investigate the hemodynamic impact of apelinergic infusion in a sheep model of septic shock, and to assess the reactivity of the endogenous apelin system and its related enzymatic degradation environment in patients with acute septic shock. This study is the first reporting a positive hemodynamic impact of APLN-13 infusion in a large animal model of septic shock and revealing simultaneous intricate homeostatic disturbances among the renin-angiotensin, kallikrein-kinin, and apelin systems involved in outcomes of acute human sepsis.

Given the therapeutic potential of adding exogenous apelinergics described in studies of sepsis in rodents^13,14^, the hemodynamic impact of administering incremental doses of APLN-13 or ELA was studied in healthy and septic sheep. Sheep are a significant large animal species with similar organ size to humans, and thus advantageous in modelling human sickness and to trial new therapies. A previous study described biphasic MAP and heart rate responses to a large intravenous (i.v.) bolus injection of APLN-13, showing that sheep were responsive to apelins^25^. Herein, although it was found that human APLN-13 and ELA bind ovine APJ, only APLN-13 acted as a positive inotrope upon infusion in healthy sheep, while ELA had cardiodepressant and hypotensive effects. These discrepant hemodynamic responses were unexpected based on previous studies reporting similar effects of ELA and APLN-13 in rodents^13,26^. Compared with that of APLN-13, the structure–activity relationship of ELA related to reduced APJ-dependent activation of G_αi1_ and recruitment of β-arrestin-2 could explain, at least in part, the different hemodynamic profiles observed in sheep. These findings will need consideration with regards to future development relating to human disease.

Beyond being efficient in healthy condition, candidate apelinergics must be operating in disease. The characteristics of this ovine experimental model of sepsis are consistent with past reports in sheep^27,28^ and compatible with human disease. This includes (1) compromised hemodynamics that is poorly fluid responsive; (2) an acute onset of multiple organ injury; and (3) metabolic perturbations with decreased systemic oxygen consumption. Concomitantly, circulating APLN levels but not ELA levels increased, suggesting defective paracrine release of ELA, with a contrasting decrease in APLN mRNA expression in the cardiorenal tissue axis. Of note, ELA mRNA expression was not detected in these ovine tissues, in contrast with the specific and almost exclusive renal expression of ELA reported in rats^29^. However, the plasma stability of APLN-13 and ELA was unaffected, and the low level of enzymatic degradation is probably related to the short-timeframe design of this ovine model.

According to evidence from this study and the literature*,* APLN-13 infusion is effective in improving cardiorenal functions without significant chronotropic changes but with a favorable cardiac energy balance and protection; and could also act as a levosimendan-like inotrope with additional anti-inflammatory; calcium sensitizing and antioxidative properties^13,30^. In contrast, while ELA showed interesting and promising hemodynamic effects with improved fluid homeostasis in a model of peritonitis in rats^13^, no beneficial effect was observed in the present setting. This highlights that the apelin system is complex and how these findings cannot be easily applied across species.

Physiological regulation of fluid homeostasis is also a critical event in hemodynamic stability and is a matter of balance between the apelin and vasopressin systems^31^. A direct counteracting effect of APLN on the antidiuretic activity of AVP can be observed in collecting ducts, modulating the docking of aquaporin-2 (AQP-2) and thus enhancing urinary output^32^. Herein, increased diuresis and improved creatinine clearance were also observed with APLN-13 infusion in septic sheep, confirming the reported effects regarding sepsis-induced reversal of blood AVP elevation, improved fluid clearance, and a negative fluid balance in rodents^13^. Overall, the APLN-13-driven beneficial effects on kidney function in sepsis seem to be closely related to its physiological interaction with the vasopressin system. This specific network is thus an important link that should be considered in future studies because fluid homeostasis and kidney function are both key issues in acute sepsis.

Whether the observed responsiveness of the apelin system in human sepsis and septic shock is appropriate is, however, unclear. Previously, we were the first to report elevated APLN-13 blood concentrations in septic shock patients, as well as in critically ill nonseptic patients^16^. In this study, considering all isoforms and degradation moieties, the increase in APLN-13 blood concentrations was higher than that previously reported, averaging more than threefold the concentrations documented in healthy volunteers, which rivals the fold increase in catecholamine levels in a similar context^33–35^. Notably, quantified ELA blood concentrations showed a smaller 1.5-fold increase in septic patients. For unclear reasons, one-fourth and one-fifth of the septic patients in this cohort remained “unreactive”, with normal ranges of circulating APLNs and ELA, respectively. This could mean that patients should be screened to select those most likely to benefit from an apelinergic therapy.

A comprehensive analysis of apelinergic blood levels requires examination of the breakdown environment. Activated RAS and KKS proteases have been associated with both accelerated protein/peptide degradation and microvascular dysfunction with multiple organ failure in sepsis and septic shock^36,37^. While the process of ELA enzymatic degradation remains essentially unknown, biologically less active or fully inactive APLN moieties can be generated by RAS- and KKS-dependent proteolysis through ACE2, NEP and KLK1 hydrolysis^20^. Indeed, exogenous APLN-13 or ELA incubated with septic plasma displayed a further shortened half-life ex vivo. Prehospital use of ACE1 inhibitors has been linked to less need for vasopressor administration and better outcomes in septic shock-may be through higher levels of APLN-13 before the onset of sepsis^38,39^. However, ACE1 blockade may be associated with sepsis-induced endothelial dysfunction and the resulting AngII deficiency related to refractory shock and impaired outcomes^40^.

Under such conditions, an alternative bypass toward a nonclassical RAS pathway producing Ang1–7 under predominantly NEP-driven activity has been suggested^41^. A bloodstream phenotype of RAS activation (*i.e.*, high ACE1/ACE2 ratio and NEP activity), along with Ang1-7 release, was found in human sepsis. Moreover, KKS has been described as a specific node linking the inflammatory and coagulation responses to microbial infection and thus contributing to the pathogenesis of sepsis^42^. The biological efficiency of the apelin system is therefore compromised under this environmental pressure, given the very short half-lives of endogenous APLNs^43^. Accordingly, a PCA of this cohort revealed that nonsurvivors were widely located within a component corresponding to enhanced apelinergic release-degradation system activity. Based on these results, we hypothesize that endogenous apelinergic system instability related to sepsis-induced dysregulation of KKS and RAS could influence the natural history and outcome of this disease.

Several limitations of this work must be mentioned. First, the large animal model selected was ovine, which was found to have defective kidney ELA expression, and reduced signaling efficacy/potency upon human ELA binding to sheep APJ was observed. Septic shock was induced by peritonitis (which was not the primary cause of sepsis in the human cohort), and the experimental period was not long enough to trigger enzymatic degradation systems. A long-term animal model of sepsis including more hemodynamic support, antibiotic therapy, and outcome assessment would offer additional distinctive advantages with more accurate and extended clinical readouts. Additionally, the human data were observational, were derived from a relatively small sample size, and were imperfectly matched for sex, and need further confirmation in a larger cohort.

## Conclusion

The apelin system is complex, with confirmed variability in cardiovascular effects across species^44^. Clearly efficient in experimental settings, this system is reactive in real-life human disease but highly susceptible to increased specific enzymatic degradation activities. From this standpoint, apelin system disturbance could influence the severity of sepsis and septic shock, opening doors for future explorative studies. The APLN-13/APJ axis thus represents a novel candidate pathway to manage hemodynamics in septic shock and could be an alternative to inotropic drugs but preventing specific enzymatic degradation or designing apelinergics resistant to degradation should be explored.

## Methods

### Experimental study design

#### Sheep instrumentation and animal model exploration

This study was approved by our institutional ethics review board (CFPA: Comité Facultaire de Protection des Animaux, #023-18BR). Thirty-six male Canadian Arcott sheep (33 ± 3 kg, 3 to 4 months old;) were included in this study according to the Animal Research: Reporting of In Vivo Experiments (ARRIVE) guidelines and received care in compliance with the Canadian Council of Animal Care and National Institutes of Health (NIH) guidelines^45^. Animals were housed for at least 48 h before the experiment in dedicated accommodations and bedding.

The day before surgery, autologous stool was retrieved for treatment of the sheep selected for fecal peritonitis induction and mixed overnight in 0.9% normal saline (NS) and 5% D-glucose at 37 °C. The animals were fasted overnight. On the morning of the day of surgery, sheep received premedication to ease animal handling and intubation (i.e., intramuscular injections of 0.1 mg/kg atropine and 10 mg/kg ketamine) before routing to the operating room for insertion of an external jugular vein catheter and administration of a 500 mL bolus of Ringer’s lactate (RL). Sheep were then continuously infused with RL (3 mL/kg/h) and 5% D-glucose (2 mL/kg/h). Tracheal intubation (7–8 Fr) was performed in a caddy under isoflurane (1–2%) mask inhalation and by an anterior approach. Sheep were then placed in the supine position on heating pads before ventilator connection (Aestiva 15, Datex-Ohmeda, USA). The initial parameters (Vt: tidal volume, 200–400 mL; P_insp_: total inspiratory pressure, + 12–20 cm H_2_O; RR: respiratory rate, 12/min) were adjusted to obtain an end-tidal CO_2_ of 35–50 mmHg (Capnomac Ultima, Datex-Ohmeda USA), and the FiO_2_ was adjusted for sublingual SpO_2_ ≥ 92%. A Levin catheter was inserted into the ovine rumen for continuous removal of excessive secretions. General anesthesia and animal comfort were ensured with a combination of titrated isoflurane (0.5%-1.5%, Baxter) and continuous i.v. infusion of ketamine (2 mg/kg/h) and rocuronium (0.2 mg/kg/h) to achieve a Sedation-Agitation Scale (SAS) score of 1. Once mechanical ventilation/oxygenation was established, a surgical team performed sequential catheter insertions: (1) on the right: (i) arterial femoral access (Pulsiocath 4Fr thermodilution catheter, 16 cm PiCCO®, Pulsion Medical System, Munich, Germany) and (ii) central venous internal jugular access (Swan-Ganz 7Fr 4 lm thermodilution catheter, Edwards Lifesciences, Toronto, Canada); (2) on the left: (i) venous femoral access (18G, dedicated to subsequent stepwise infusions) and (ii) arterial carotid-to-LV access (SPR-350 pressure catheter, Millar Inst Oakville, ON, Canada); (3) ultrasound-guided urinary bladder catheter (#8.3F pigtail, Cook Medical, Bloomington, IN, USA); and (4) two intraperitoneal (i.p.) 28-to-34Fr chest tubes (one posterior, one anterior).

Baseline hemodynamic parameters and vital sign measurements (HR: heart rate, MAP: mean arterial pressure, SVR: systemic vascular resistance, CVP: central venous pressure, PAOP: pulmonary artery occlusion pressure and wedge pressure), dP/dt max and min, LV end-systolic pressure (LVESP), CO, stroke volume (SV), GEDV, and extravascular lung water (EVLW) were then recorded. Blood temperature, baseline central venous O_2_ saturation (ScvO_2_), and arterial blood gas (RapidLab® 348, Siemens Healthcare Diagnostics, Montreal, Qc, Canada) and lactate levels (Lactate Plus, Nova Biomedical, Waltham, MA, USA) were also monitored. A slightly modified sheep SOFA^46^ score was calculated after reached shock criteria defining the “shock” time point and before euthanasia named the “final” time points, defined as a MAP below 65 mmHg for level 1 of the cardiovascular system assessment and assuming a constant level 3 of the central nervous system (Glasgow score 6–9) at “shock”. Additional blood sample were collected at the “shock” and “final” endpoints for the biomolecular measurements. All blood test and rumen secretion removal volumes were estimated and systematically balanced by compensatory NS infusion.

After 2 g/kg autologous stool slurry was injected into both intra-peritoneal (i.p.) tubes, RL and 5% D-glucose infusions were stopped, and all the above parameters were monitored every 30 min. Serial blood cultures (Signal Blood Culture System, Thermo Scientific™ BC0100M, USA) were performed to assess whether the septic shock criteria were met in the sheep and to document and specify active bacterial translocation. All five criteria for experimental septic shock had to be met before implementing the next interventional steps. These criteria, measured from baseline, included (1) a decrease in MAP to < 60 mmHg or ≥ 40%, (2) a decrease in CO to < 3.5 L/min/m^2^ or ≥ 40%, (3) less than 1.5 mL/kg/h urine output, (4) ScvO_2_ below 70%, and (5) arterial lactate content above 2 mmol/L or a threefold increase. At this time point, hemodynamic parameters were recorded, and blood tests were performed.

A 30 mL/kg i.v. bolus of balanced crystalloid RL for resuscitation was infused for 1 h (as recommended by the 2018 Surviving Sepsis Campaign guidelines)^47^ before restarting the initial infusion, RL (3 mL/kg/h) and 5% D-glucose (2 mL/kg/h). Sheep were randomly and blindly assigned to an incremental five-step dose–response infusion procedure (20 min) using e-syringes containing a solution of the apelinergic APLN-13 or ELA in NS (d1: 0.025, d2: 0.25, d3: 2.5, d4: 6.25, d5: 12.5 nmol/kg/h), or NS alone. Oxygen consumption (VO_2_) was calculated as VO_2_ = CO x (CaO_2_-CvO_2_) × 10 with Ca or vO_2_ = (Hb × 1.34 x SaO_2_) + (PaO_2_ × 0.003) at the baseline, shock, and fluid resuscitation time points and for every assessed dose of tested article until the endpoint. Hemodynamics, arterial blood gases and lactate content and necessary blood or clinical parameters for SOFA score calculation were recorded at all dose steps. At the end of the agonist dose–response trial Sheep were euthanized (i.v. injection of 90 mg/kg phenobarbital) before sampling lung, heart, and kidney tissues.

#### Ovine biochemical assays

Ovine plasma and urine concentrations of several additional compounds were quantified with commercially available ELISA kits (My BioSource, San Diego, CA, USA): troponin T (Tn T) (MBS068213; detection range: 31.2–1000 pg/mL; sensitivity: 5 pg/mL), arginine vasopressin (AVP) (My Biosource; MBS9351668; detection range: 3.12–100 pg/mL; sensitivity: 1 pg/mL), catecholamines (My Biosource; MBS751915; detection range: 1–2,500 pg/mL; sensitivity: 1 pg/mL), and cortisol (My Biosource; MBS706097; detection range: 0.049–200 ng/mL; sensitivity: the lowest concentration differentiated from zero) kidney injury molecule-1 (KIM-1) (My Biosource; MBS2503588; detection range: 31.25–2,000 pg/mL; sensitivity: 18.75 pg/mL). Plasma and urine creatinine levels were measured with a chemistry analyzer (Element DC, Heska, CO, USA), and creatinine clearance was calculated as previously described ^48^. Enzymatic degradation activities in plasma were evaluated at the experimental “final” endpoint, as detailed in the Common Methods section.

#### Apelinergic system expression and pharmacology

##### Quantitative reverse-transcription polymerase chain reaction

RNA was extracted from heart and kidney tissues with a RNeasy Mini Kit (Qiagen, Germany). iScript Reverse Transcription Supermix (Bio-Rad) was used to prepare cDNA from 1 μg of total RNA in 20 μL. Real-time PCR was performed on technical duplicates with cDNA diluted 30 × in nuclease-free water and using SsoAdvanced™ Universal SYBR® Green Supermix (Bio-Rad, CA, USA) in a Mastercycler® ep RealPlex (Eppendorf, On, CA). Relative expression was normalized to the average expression of the *Gapdh* and *Actb* housekeeping genes using the 2(-ΔCt) method. Sequences of the primers (forward: Fwd; reverse: Rev) used to determine the expression level of target genes were: *Apln*, Fwd: CTTCTGACGGGAAGGAGATG, Rev: CGGAACTTCCTCCGACCT, *Aplnr*, Fwd: TTGTGGGTCTGGAGGGTAAG, Rev: GCTGGGAGCATTTCAGAGAC; *Gapdh*, Fwd: TGGTGAAGGTCGGAGTGAACGG, Rev: TGAAGGGGTCATTGATGGCAACG; *Actb*, Fwd:CCTTAGCAACCATGCTGTGA, Rev: AAGCTGGTGCAGGTAGAGGA.

##### Sheep APJ plasmid cloning

The sequence of the apelin receptor was synthetized with gBlocks Gene Fragments (Integrated DNA Technologies, USA) and subcloned into pEYFPC1 (a modified vector in which we replaced the YFP sequence with that for two HA tags) with an NEBuilder HiFi DNA Assembly Cloning Kit (New England Biolabs, MA, USA). The *Ovis aries* APJ sequence was obtained from the RefSeq database (XM_027979777.1).

##### Radioligand binding

Competitive radioligand binding experiments were performed as previously described^43^ using [^125^I]-(pyr^1^)Apelin(APLN)-13 (820 Ci/mmol) prepared with IODO-GEN (1,3,4,6-tetrachloro-3a,6a-diphenyl-glycoluril; Thermo Scientific Pierce, Canada)^49^. In brief, HEK293 cells with surface expression of HA epitope-tagged sheep APJ generated as previously described (ref Murza) were incubated with 0.2 nM radiolabeled APLN-13 and increasing concentrations of cold APLN-13 or ELA synthesized as previously described^50^ (10^–11^ to 10^–5^ M) for 1 h at room temperature. The γ emission was quantified using a γ-counter 1470 Wizard from PerkinElmer (Waltham) (80% efficiency). Nonspecific binding, measured in the presence of 10−5 M unlabeled APLN-13, did not exceed 5% of total signal. All binding data were calculated and plotted using GraphPad Prism 8 (La Jolla, CA) and represent the mean ± SEM of three replicates.

##### Bioluminescence resonance energy transfer (BRET) assays for G_αi1_ activation and β-arrestin-2 recruitment

BRET experiments were performed as previously described^10^. In brief, HEK293 cells were transfected with plasmids encoding sheep APJ, Gαi1-RlucII (inserted at residue 91), GFP10-Gγ2, and Gβ1 (from cDNA.org) (for the BRET-based Gαi1 activation assay) or encoding sheep APJ-GFP10 and RlucII-β-arrestin2 (for the BRET-based β-arrestin-2 recruitment assay)^50^. To perform the BRET assays, 50,000 cells/well were transferred into white 96-well plates (BD Bioscience, Mississauga, Canada) and incubated at 37 °C overnight. The cells were then washed with PBS and stimulated with APLN-13 or ELA (10^−5^ to 10^−11^ M) at 37 °C for 5 min (Gαi1) or 30 min (β-arrestin-2). After stimulation, 5 μM coelenterazine 400A was added to each well, and the plate was read on a GeniosPro plate reader using the BRET2 filter set (Tecan, Austria). The BRET2 ratio was calculated as GFP10em/RlucIIem.

### Patient study design

This was a prospective observational nonregistered study of a cohort of patients admitted to the intensive care unit (ICU) at the Centre Hospitalier Universitaire de Sherbrooke (CHUS, QC, Canada) for sepsis or septic shock. The study was approved by our local ethics review board (Comité d’Éthique de la Recherche -CÉR- du CIUSSS de l’Estrie-CHUS, #2-18-476 12-125). This study was conducted in accordance with the guidelines of the Declaration of Helsinki. Patients or their relatives provided written informed consent.

#### Recruitment

Consecutive patients who were determined to have sepsis or septic shock on admission according to the new Sepsis-3 definition of sepsis^51^ were enrolled. Study participants were age-matched to healthy volunteers recruited through the end of 2019, resulting in an ~ 2:1 sick-to-healthy ratio. Blood samples were drawn once in the morning and again during the first 36 h of ICU admission for participants. All therapies were managed at the discretion of the treating physician. The severity of illness at baseline was calculated on admission using the APACHE II and SOFA scores. Clinical parameters included 28-day mortality. Refractory septic shock was defined as the need for high-dose vasopressor support greater than 0.5 μg/kg/min norepinephrine or equivalent, despite appropriate fluid resuscitation and 300 mg/day i.v. hydrocortisone or equivalent. Since several frequently prescribed drugs have been documented to affect apelin blood levels, patient medical records were specifically reviewed to identify patients currently taking statins, glibenclamide/glyburide, gliclazide, rosiglitazone, spironolactone, gliptins, and metformin or combinations thereof, as well as Entresto® (sacubitril + valsartan), which contains an NEP inhibitor. However, drug history was not a ground for exclusion.

#### General blood cyto-biochemical measurements

Human arterial blood was collected in EDTA-containing tubes, and a small portion was dedicated to whole blood cell and polymorphonuclear (PMN) cell counting (10^9^/L) with band-cell (%) determination using a DxH 900 hematology analyzer de Beckman Coulter. The remaining sample was used for PMN isolation using Polymorphprep® (Axis-Shield Diagnostics Ltd., Dundee, Scotland) density centrifugation according to the manufacturer’s instructions. Isolated PMN cells were suspended in RPMI medium at a concentration of 1 × 10^6^ cells/mL, washed in PBS, and incubated for 30 min on ice with the fluorochrome-conjugated monoclonal antibodies anti-CD64-APC/Cy7 (clone 10.1) and anti-CD66b-PerCP/Cy5.5 (clone G10F5) (BioLegend, San Diego, CA, USA). The cells were then fixed in 2% paraformaldehyde (BD Cytofix™, BD Pharmingen, USA) for 10 min at room temperature. Isotype controls were used to quantify the nonspecific background signal. Cells were washed twice in PBS prior to measurement with a FACSCanto instrument. PMN purity was first assessed using expression of the marker CD66b higher than 98%, and PMN CD64 expression expressed as the mean fluorescence intensity (MFI).

Blood levels of pentraxin 3 (PTX3) were measured using high-sensitivity cytokine magnetic bead assays (Milliplex® MAP Multiplex Assays, EMD Millipore, Billerica, MA, USA) according to the manufacturer’s instructions. Data were analyzed using Multiplex Assay Analysis Software (EMD Millipore) and are expressed in ng/mL.

The angiotensin 1–7 (Ang1-7) plasma concentration was quantified with a commercially available ELISA kit and performed according to the manufacturer instructions (Abbexa; abx251960; Houston, TX, USA; range of 15.625–1,000 and a sensitivity of 9.38 pg/mL).

Arterial lactate (mmol/L) was assessed in septic patients as described previously^52^.

Human blood cortisol content was measured with the ADVIA Centaur system (Siemens Medical Solutions Diagnostics, Tarrytown, NY, USA) with a sensitivity of 5.5–2,069 nmol/L and a range of 85–618 nmol/L.

#### Apelinergic enzymatic degradation activities in plasma

##### Neprilysin (NEP)

As previously described^53^, 20 µL of plasma, 10 µL of substrate (5 mmol/L glutaryl-Ala-Ala-Phe-AMC; Peptides International, Louisville, KY, USA) and 50 µL of assay buffer (0.1 mol/L Tris–HCl, pH 7.6) were incubated at 37 °C for 30 min. The reaction was stopped by adding 10 µL of the NEP inhibitor phosphoramidon (0.1 mmol/L; Sigma, Oakville, ON, Canada) and incubating the samples on ice. Background controls were processed in the same manner except that phosphoramidon was added before the incubation at 37 °C. In the next step, the samples were incubated at 37 °C for 30 min with 10 µL of aminopeptidase M (500 mg/L, EMD Millipore, Etobicoke, ON, Canada) and 5 mmol/L EDTA. The reaction products were diluted in 3 mL of assay buffer, and the fluorescence was measured using a Tecan Infinite M1000 plate reader at an excitation wavelength of 360 nm and an emission wavelength of 440 nm. NEP activity was calculated from the difference between the sample (S) and control (C) values with the equation (S—C)/194^53^.

##### Angiotensin converting enzyme 2 (ACE2)

The assay to measure ACE2 activity was performed as previously described (10); briefly, 2 µL of plasma was incubated with 100 µl of buffer (100 mM Tris–HCl, 600 mM NaCl, 0.5 mM ZnCl_2_, pH 7.5) and 2 µl of 1 mM quenched fluorescent substrate (Mca-Ala-Pro-Lys (Dnp)-OH; Enzo Life Sciences, Farmingdale, NY, USA) at 37 °C for 16 h. Fluorescence was measured at 405 nm with excitation at 320 nm. The results were expressed as relative fluorescence units (RFU)/µL of plasma/h.

##### Angiotensin converting enzyme 1(ACE1)

The assay to measure ACE1 activity was performed as previously described^54^; briefly, 0.83 µL of plasma was incubated at 37 °C for 25 min with 73 µL of assay buffer (0.5 M borate, 15.63 mM ZnCl_2_, 5.45 M N-hippuryl-His-Leu; Sigma). Fluorescence was measured at an excitation wavelength of 355 nm and an emission wavelength of 535 nm. The results are expressed as RFU/µL of plasma.

##### Kallikrein (KLK1)

The plasma KLK1 activity was quantified with a commercially available fluorescence assay kit and performed according to the manufacturer instructions (Anaspec; AS-72255; Fremont, CA, USA). Fluorescence was measured at an excitation wavelength of 490 nm and an emission wavelength of 520 nm. Results were expressed as relative fluorescent units/minute.

### Common methods

#### Apelinergic concentrations in plasma

Plasma was obtained from EDTA-containing blood samples collected from included patients and sheep by centrifuging at 1,600 × *g* for 10 min at 4 °C. Additional extraction on a C18-E SEP column (Phenomenex, Torrance, CA, USA) with overnight freeze-drying was mandatory for samples used for ELA measurements. APLN-36, APLN-17, APLN-16, APLN-13, and APLN-12 plasma concentrations, as well as those of the corresponding shorter degradation products, were measured with a specific commercially available ELISA kit (LifeSpan BioSciences; LS-F25717; WA, USA; detection range: 31.25–2,000 pg/mL; sensitivity: less than 18.75 pg/mL). ELA was quantified using a commercially available ELISA kit (Peninsula Laboratories International; S-1508; CA, USA) with a measurement range of 0–100 ng/mL.

#### Aperlinergic stability in plasma

As previously described^43^, 27 μL of plasma mixed with 6 μL of a 1 mM aqueous solution of APLN-13 or ELA were incubated at 37 °C. Proteolytic degradation was stopped by adding 140 μL of 50% acetonitrile, 50% ethanol, and 0.25 mM N, N-dimethylbenzamide at 0, 0.5, 1, 2, 4, or 6 h. The reaction mixture was filtered by centrifugation at 2,000 rpm with Impact Protein Precipitation Filter plates (Phenomenex, CA, USA). Next, the reaction mixture was diluted with 80 μL of water and analyzed overnight with an Acquity class H ultra-performance liquid chromatography-mass spectrometry (UPLC-MS) system from Waters (Milford, MA, USA) (column: Acquity UPLC CSH C18, 2.1 mm × 50 mm, packed with 1.7 μm particles). Results were expressed as a percentage of the initial zero-time spike area.

##### Sample size calculation

Based on a pilot assessment of sheep with CO as the primary outcome, a difference of 1 L/min was considered “clinically significant”, with a targeted sample size of at least 6 animals/group to reach an α of 0.05 and a power of 80%.

##### Data analysis

Results are expressed as the mean ± SEM (or SD) or median [25–75th percentiles], when appropriate. Normality was assessed with the D’Agostino and Pearson test. Comparisons between healthy volunteers and septic patients were performed with the Mann–Whitney U test. Linear regression was used for dependent variable analyses. All data were calculated and plotted using GraphPad Prism 8 (La Jolla, CA). Principal component analysis was conducted with R software (v.3.6.1). Figures were produced with the FactoMineR package and ggplot2. Quantitative variables were compared with a paired Student’s *t*-test or a Mann–Whitney U test. Dose–response and time-course analyses were performed with repeated measures two-way ANOVA followed by Tukey's multiple comparison test. P < 0.05 was considered significant.

## Supplementary Information

Supplementary Information.

## Abbreviations

ACE 1 & 2: Angiotensin converting enzymes 1 & 2
Ang1-7: Angiotensin1-7
APACHE II: Acute physiology and chronic health evaluation II
APLNs: Apelins
AQP-2: Aquaporin-2
AVP: Vasopressin
ELA: Elabela
EVLW: Extra vascular lung water
GEDV: Global end-diastolic volume
HR: Heart rate
ICU: Intensive care unit
KLK1: Kallikrein
KKS: Kallikrein Kinin system
KIM-1: Kidney injury molecule-1
LV: Left ventricle
LVESP: Left ventricle end systolic pressure
MFI: Mean fluorescence intensity
NEP: Neprilysin
PAOP: Pulmonary Artery Occlusion Pressure
PCA: Principal Component Analysis
PEI: Polyethylenimine
PMN: PolyMorphoNuclear
PTX3: Pentraxin 3
RAS: Renin angiotensin system
RL: Ringer’s Lactate
SAS: Sedation agitation scale
ScvO2: Central venous saturation of oxygen
SV: Stroke volume
SVR: Systemic vascular resistance
SOFA: Sequential organ failure assessment
