# Supplementary material for: Apelin-13 in septic shock: effective in supporting hemodynamics in sheep but compromised by enzymatic breakdown in patients
Source: Sci Rep. 2021 Nov 23;11:22770. doi: 10.1038/s41598-021-02087-4 (PMC8611018; doi:10.1038/s41598-021-02087-4)
Supplement: Supplementary file 1 — Supplementary Information. [file 41598_2021_2087_MOESM1_ESM.doc]

**Apelin-13 in Septic Shock: Effective in Supporting Hemodynamics in Sheep but Compromised by Enzymatic Breakdown in Patients**

David Coquerel,^1, 7^ Julie Lamoureux,^1, 3, 7^ Frédéric Chagnon,^1,7^ Kien Trân,^1, 5^ Michael Sage,^4^ Etienne Fortin-Pellerin,^1, 4^ Eugénie Delile,^1, 7^ Xavier Sainsily,^1, 2, 5^ Justin Fournier, ^1, 2, 5^ Audrey-Ann Dumont,^2^ Mannix Auger-Messier,^1, 2, 6^, Philippe Sarret,^1, 5, 6^ Eric Marsault^†^,^1, 5, 6^ Jean-Paul Praud,^1, 4^ Tamàs Fülöp,^3^ and Olivier Lesur^1, 7*^

^1^ Centre de Recherche Clinique du CHUS

^2^ Département de Médecine, Service de Cardiologie

^3^ Centre de Recherche sur le Vieillissement

^4^ Départements de Pédiatrie et de Pharmacologie/Physiologie

^5^ Département de Pharmacologie-Physiologie

^6^ Institut de Pharmacologie de Sherbrooke (IPS)

^7^ Unité des Soins Intensifs Médicaux et Service de Pneumologie

Faculté de Médecine et des Sciences de la Santé, Université de Sherbrooke, Sherbrooke, QC, Canada

^†^: deceased

**Running title:** The Apelin System in Septic Shock.

***Corresponding author information:** Olivier Lesur MD, PhD, Department of Intensive Care Medicine, Faculty of Medicine and Health Sciences, University of Sherbrooke, 3001 12^th^ Avenue Nord, Sherbrooke, J1H 5N4, Quebec, Canada. Phone: 819 346-1110 ext. 15406. E-mail: [Olivier.Lesur@USherbrooke.ca](mailto:Olivier.Lesur@USherbrooke.ca)

**SUPPLEMENTARY FIGURES**

**
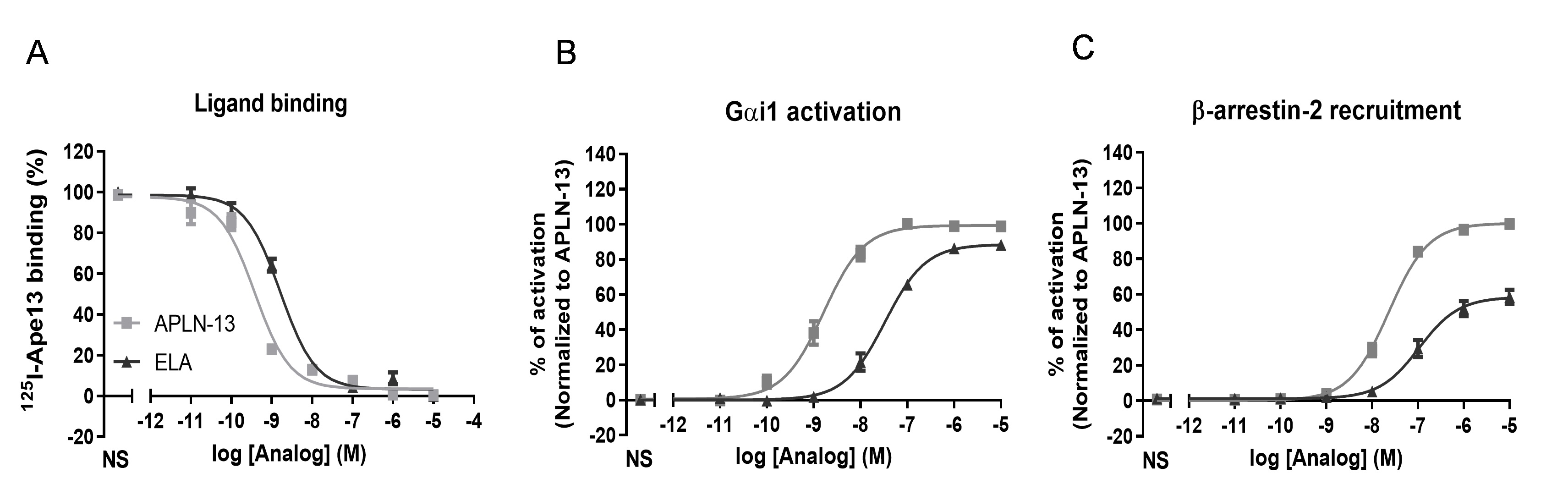
**

**Fig. S1 Elabela (ELA) and apelin-13 (APLN-13) exhibit distinctive signaling after apelin receptor APJ binding.**

**(A)** Competition binding curves of human APLN-13 (n=3) and ELA (n=3). Concentration-response relationships of APLN-13 (n=3) and ELA (n=3) pertaining to their ability to engage Gαi1 **(B)** and to recruit β-arrestin-2 **(C)**. Assays were performed with HEK293 cells expressing sheep APJ on the cell membrane. ELA binding to sheep APJ displayed lower potency in G_αi1_ activation and lower potency and efficacy in β-arrestin-2 recruitment than APLN-13 binding. All data represent the mean ± SEM of three determinations.

#
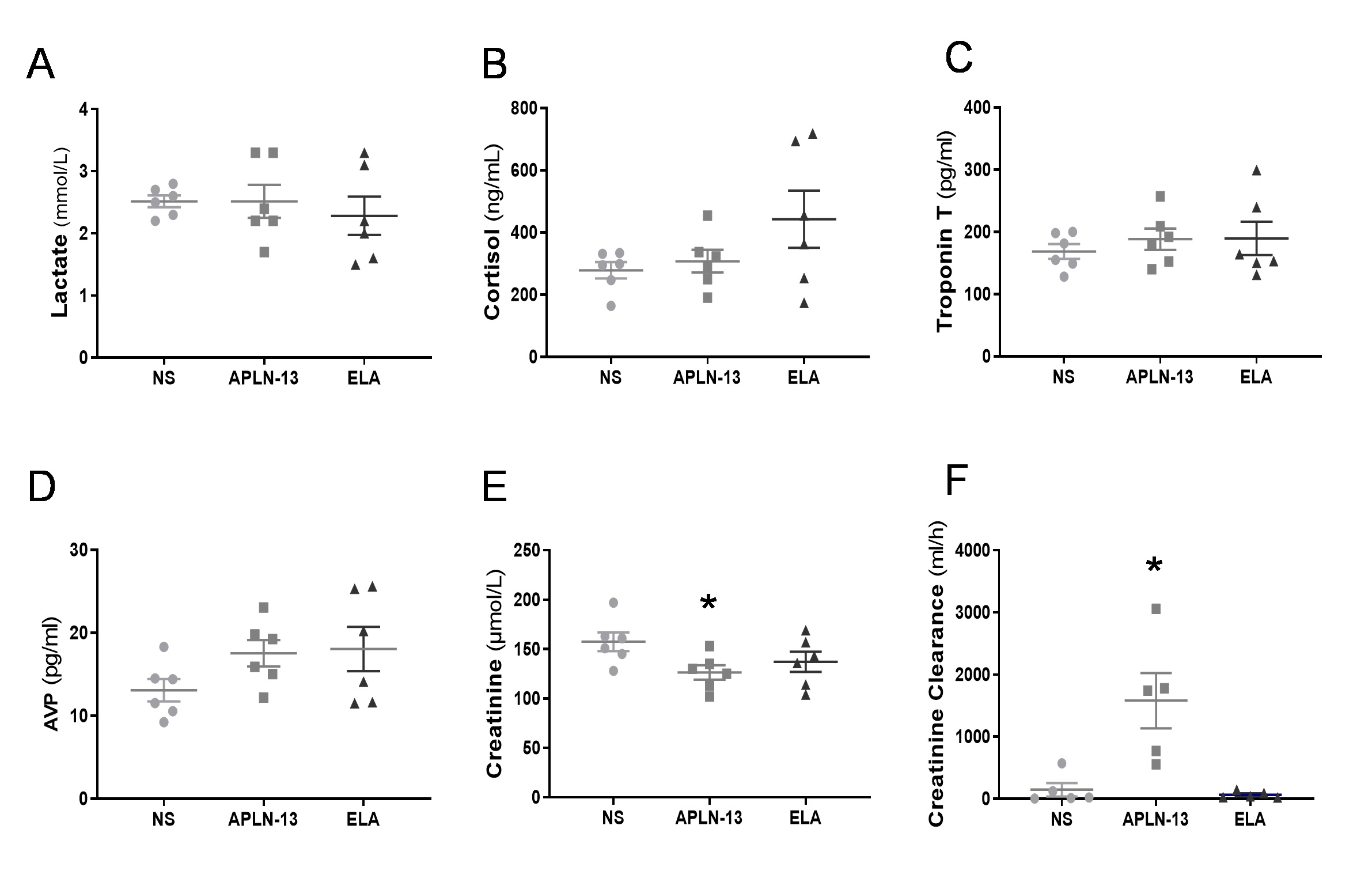


# Fig. S2: End-point biomarkers of neuro-hormonal activation and multi-organ failure in ovine fecal peritonitis (FP)-induced septic shock.

Arterial levels of lactate **(A)**, cortisol **(B)**, troponin T (Tn T) **(C)**, arginine-vasopressin (AVP) **(D)**, creatinine **(E)** and calculated creatinine clearance **(F)**, altogether assessed at the end of last d5 apelin-13 (APLN-13), elabela (ELA) or normal saline (NS) infusion (n = 6/group). APLN-13 tended to improve kidney function. Results are expressed as mean ± SEM. *p < 0.05 vs. NS, one-way ANOVA test.


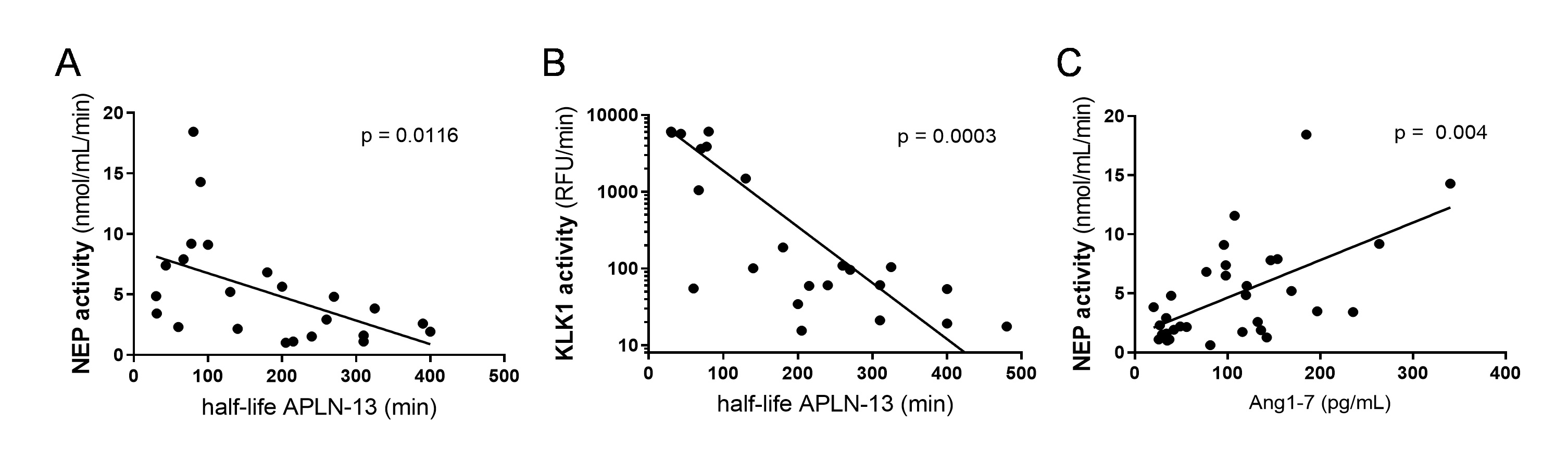


**Fig. S3: Relationships between plasmatic KLK1 and NEP enzymatic activities, and angiotensin1-7 (Ang 1-7) levels in patients with sepsis/septic shock (Sepsis).**

**(A and B)** NEP and KLK1 enzymatic activities and relationship to the calculated half-life of exogenous APLN-13 in plasma from patients with sepsis/septic shock (n =33), analyzed by linear regression (R^2^ = 0.27 and 0.72, respectively). The higher the NEP and KLK1 activities the lower the half-life of APLN-13 **(C)** Association between neprilysin (NEP) activity and Angiotensin 1-7 (Ang1-7) levels (R^2^ = 0.34) in plasma from patients with sepsis/septic shock (n =33). Ang 1-7 levels did not correlate with Angiotensin Converting Enzyme 2 (ACE2) activity (data not shown) but with NEP activity in this context. All data were analyzed by linear regression.
